# Primary and Secondary Rewards Differentially Modulate Neural Activity Dynamics during Working Memory

**DOI:** 10.1371/journal.pone.0009251

**Published:** 2010-02-16

**Authors:** Stefanie M. Beck, Hannah S. Locke, Adam C. Savine, Koji Jimura, Todd S. Braver

**Affiliations:** 1 Department of Psychology, Washington University in St. Louis, St. Louis, Missouri, United States of America; 2 Department of Psychology, Dresden University of Technology, Dresden, Germany; University College London, United Kingdom

## Abstract

**Background:**

Cognitive control and working memory processes have been found to be influenced by changes in motivational state. Nevertheless, the impact of different motivational variables on behavior and brain activity remains unclear.

**Methodology/Principal Findings:**

The current study examined the impact of incentive category by varying on a within-subjects basis whether performance during a working memory task was reinforced with either secondary (monetary) or primary (liquid) rewards. The temporal dynamics of motivation-cognition interactions were investigated by employing an experimental design that enabled isolation of sustained and transient effects. Performance was dramatically and equivalently enhanced in each incentive condition, whereas neural activity dynamics differed between incentive categories. The monetary reward condition was associated with a tonic activation increase in primarily right-lateralized cognitive control regions including anterior prefrontal cortex (PFC), dorsolateral PFC, and parietal cortex. In the liquid condition, the identical regions instead showed a shift in transient activation from a reactive control pattern (primary probe-based activation) during no-incentive trials to proactive control (primary cue-based activation) during rewarded trials. Additionally, liquid-specific tonic activation increases were found in subcortical regions (amygdala, dorsal striatum, nucleus accumbens), indicating an anatomical double dissociation in the locus of sustained activation.

**Conclusions/Significance:**

These different activation patterns suggest that primary and secondary rewards may produce similar behavioral changes through distinct neural mechanisms of reinforcement. Further, our results provide new evidence for the flexibility of cognitive control, in terms of the temporal dynamics of activation.

## Introduction

A remarkable feature of human cognitive behavior is the ability to act in an intelligent, goal-directed manner. Such goal-directed actions are thought to be the result of a variety of cognitive control processes that enable the formation, maintenance, and updating of goal representations, as well as top-down biasing mechanisms that enhance the processing of goal-relevant information, inhibit goal-irrelevant information, and detect conflicts between them. An important aspect of these control mechanisms, that is nevertheless often overlooked, concerns the role of non-cognitive factors such as affect and motivation. In particular, the pursuit of goals must be prioritized by the value of the outcomes attached to them. Indeed, it seems clear that a primary function of affect and motivational systems is to provide just these sorts of value and prioritization signals. Thus, the cognitive control processes that regulate and coordinate goal-directed behaviors are likely to interact with brain systems that determine the potential affective/motivational value of possible responses [1]. The current study addresses this issue by focusing on the effect of different motivational variables on brain activity during cognitive task performance.

It is not surprising that there has been a growing interest within cognitive neuroscience on how executive or cognitive control processes might interact with motivational states and the processing of reward information. A few studies have begun to investigate how manipulations of reward and motivational states modulate neural activity during the performance of different cognitive tasks [1]–[4]. A common finding in these studies has been that reward/motivational manipulations increase activation in a network of brain regions that are typically engaged by executive control demands during task performance, such as the lateral prefrontal cortex (PFC) and parietal cortex. Additionally, this work has revealed that motivational manipulations also tend to engage regions that are considered to be more closely linked with reward and affective processing, such as the striatum, orbitofrontal cortex, and amygdala [5].

However, an important question that still has not been well addressed in the previous literature is the temporal dynamics by which motivational effects influence executive control. Executive control processes can be sustained in nature, reflecting maintained goal states or expectancies, or transient, reflecting moment-to-moment fluctuations in environmental demands, or internal processes (e.g., error detection). Likewise, motivational signals can also fluctuate transiently (e.g., high value vs. low value rewards available) or in a more state-like manner (e.g., thirst vs. satiation). Thus, it is important to implement experimental manipulations and methods that permit dissociation of executive and motivational processes according to their temporal dynamics. In fMRI studies, it is possible to decompose brain activation signals into transient and sustained effects through the use of mixed blocked/event-related designs [6], [7]. Such designs have been used to selectively dissociate distinct executive control components in a wide-range of domains, including task-switching, episodic memory, prospective memory, and decision-making. However, the mixed design has only rarely been used in studies of motivation and executive control [8], [9].

In Locke & Braver [8], it was found that task performance under reward conditions was primarily associated with a sustained rather than transient increase in activation within executive regions such as PFC and parietal cortex, when compared to performance under baseline, non-reward conditions. However, a limitation of this study was that the manipulation of reward occurred only in a blocked rather than trial-by-trial manner. Thus, it was not clear whether the sustained effects reflected non-specific processes such as arousal or attention, or even the requirement to maintain the increased reward value of the task across trials (since reward information was not explicitly provided to participants on each trial). Conversely, in prior studies that have manipulated reward only in a trial-by-trial fashion, it has not been possible to establish whether the motivational effects of potential rewards produce sustained changes in brain activity that are independent of any trial-related effects. Thus, an optimal experimental design for identifying and potentially dissociating sustained from transient motivational effects is one in which rewards are manipulated in both a block-based and trial-by-trial manner.

In the current study, we utilized such a design to examine the effects of motivation on cognitive control during a working memory task. Participants performed a standard item recognition paradigm [10], in both reward and non-reward blocks. Furthermore, within reward blocks, trials were randomly varied among three types: no-reward, low-reward and high-reward. Because the trial-by-trial manipulation of reward value was indicated to participants at the start of each trial, this value could be encoded and represented in a fully transient manner. Thus, there was no *a priori* requirement for reward information to be maintained across trials (since these were continually changing), or for the motivational effects to be tonic in nature. That is, because the reward value changed on a trial-by-trial basis, a tonic state change might be a less efficient way of adapting cognitive processing and performance. Nevertheless, we hypothesized that sustained effects on cognitive control processes might still be present, and reflect the increased incentive salience of the reward block (as a whole) relative to the no-reward block.

A second goal of the study was to address questions related to whether the type of reward available influences the nature of motivational effects. For example, motivational incentives can be categorized as involving primary rewards, such as food or liquid, or secondary rewards, such as money. Human studies examining the neural correlates of motivational effects in cognitive tasks have typically employed monetary reinforcers [2], [8], whereas in animal studies motivational effects are standardly studied with primary reinforcers, such as food or liquid. In principle, there is no reason why primary reinforcers cannot be examined in human motivation-cognition studies. Indeed, there is a growing neuroimaging literature that has employed primary rewards within the context of classical and instrumental conditioning [5]. One benefit of employing primary rewards to examine motivational effects in human studies is the ability to draw closer links to the animal work. But more importantly, such studies would provide the ability to directly test whether motivational incentives exert their effects in a domain-general or category-specific manner [11], [12]. There seems to be a basic, but implicit assumption in the literature that motivational incentives effects are category-independent [13], [14], but this assumption has not yet been directly tested. To our knowledge, there have not been any studies directly comparing the impact of different incentive categories on brain activity and behavior during performance of cognitive tasks (but see [11], [12]. for conditioning studies of this type). There are reasons to predict that there may be interesting effects of incentive type. Specifically, primary and secondary incentives might influence behavior through different neuronal routes or circuits, based on the way the reward information is encoded (e.g., primary incentives may operate primarily through sensory and subcortical pathways while secondary incentives may operate through multi-modal cortical ones).

In the current study we directly compared the effects on cognitive control processes and associated brain activity dynamics associated with reward incentives, when the rewards were monetary bonuses received after the session (Money condition) or drops of juice delivered directly on each trial (Liquid condition). A within-subjects design was employed with incentive manipulated across blocks, but all other aspects of the design and task held constant. Furthermore, in the Liquid block as well as the Money block, reward value was also manipulated on a trial-by-trial basis. Thus, our design permitted not only a direct comparison of Liquid vs. Money rewards on neural activity, but also whether the incentive category effects differentially affected sustained vs. transient activation components. Because this was the first study of its type, we did not have any strong *a priori* predictions regarding whether common or selective effects of incentive type would be observed.

## Methods

### Ethics Statement

The research protocol was approved by the Washington University Human Research Protection Office, and all participants provided written informed consent prior to participation.

### Participants

Thirty-one right-handed participants (ages 19–34, 17 female) volunteered to participate in the study in return for payment ($25/hour). All participants were recruited from Washington University, were native English speakers, and free from psychiatric or neurological disorders. Each participant was also screened for any physical or medical condition affecting eligibility for fMRI prior to being scanned.

### Task

**Figure 1:**
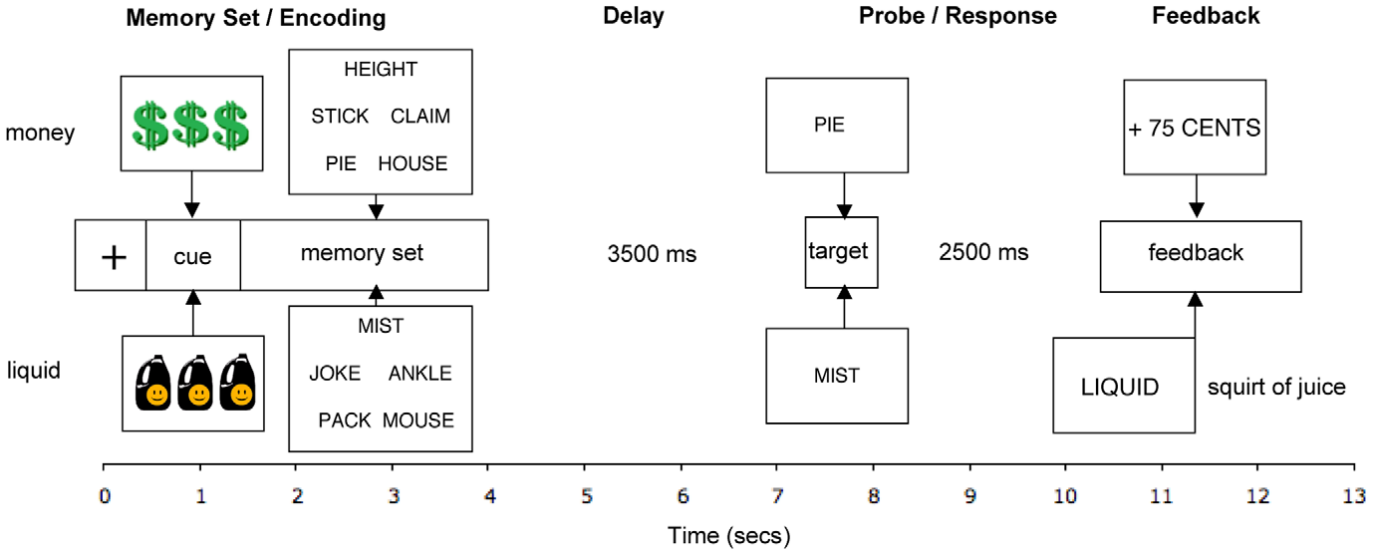
Schematic display of Liquid and Money incentive trials. Each trial started with a fixation cross (500 msec), followed by a cue indicating the type of incentive at stake (1000 msec; cues indicate high-incentive trials), a set of stimulus words (2500 msec), a delay period (3500 msec), a probe word (500 msec), a second delay (2500 msec) and a feedback phase, based on performance on the trial (2000 msec). Total duration: 12.5 seconds.

Participants engaged in a delayed item recognition of task of working memory (WM) [10], while reward incentives were manipulated across three blocked conditions. The stimuli utilized in this working memory task were eleven hundred words taken from the English Lexicon Project at Washington University (http://elexicon.wustl.edu; [15]). These words were each 1 to 2-syllables and 4–6 letters in length, and were classified as nouns, adjectives, or verbs. The mean frequency of the words was approximately log-10 based on the Hyperspace Analogue to Language (HAL) corpus [16]. Adverbs, plurals, and emotionally-valenced words were not included. Each word was presented only once during the course of the experiment. In the Baseline condition of this paradigm, no incentives were provided. In the Money condition, the incentive was an opportunity to win monetary bonuses on a subset of trials. In the Liquid condition, the incentive was the opportunity to receive (again on a subset of trials) a squirt of apple Juice delivered directly into the mouth. All conditions shared a common trial structure (see Figure 1). First, a cue was presented (1000 msec), indicating the presence and type of incentive available on that trial. Next, a memory set of 5 words was presented on the screen (2500 msec), followed by a delay period (retention interval; 3500 msec). After the delay, a probe word appeared and the participant made a manual response indicating whether or not the probe was included in the memory set. Half of the probes were targets (in the memory set) and the other half were non-targets (not in the memory set). Following the response, reward feedback was provided.

During the scanning session, the incentive conditions were performed in a blocked fashion. Participants first performed baseline blocks without any instruction that future blocks would be performed with incentives, followed by four types of incentive blocks, performed in counter-balanced order across participants: reward money, reward liquid, penalty money, and penalty liquid. There were two scanning runs per condition, for a total of 10 runs, including 2 runs of baseline. Each run lasted approximately 8.5 minutes, and consisted of 2 blocks of 10 trials, alternating with 3 fixation blocks (approximately 50 seconds each), for a total of 40 trials per condition. The penalty conditions were not the focus of the current paper, and will not be discussed further.

Within the incentive blocks, trials were divided into 3 types: no-incentive (NO: 20%), low reward (LO: 40%; Money  = 25 cents; Liquid  = 0.5 ml) and high reward (HI: 40%; Money  = 75 cents; Liquid  = 1.5 ml). The amount of liquid delivered at each trial was titrated as to be equivalent to a very small sip of liquid - enough to be discernible as a tangible amount of liquid, but small enough to be swallowed easily while laying on one's back in the scanner. Additionally, the amount was chosen to minimize chances of satiation across the experiment. On incentive trials, incentives were delivered only when performance was accurate, and reaction times were faster than a cutoff value. This cutoff value was defined individually for each participant, based on median correct trial RT during the Baseline block. On trials in which the cutoff was not met, this was indicated to participants in the appropriate sensory modality (Money: “--” visual feedback; Liquid: delivery 0.5 ml of a tasteless, saliva-like solution of KCl and NaHCO; KCl and NaHCO). On no-incentive and Baseline trials, a neutral message was provided during this time period (“Next Trial Coming Up”). To ensure that participants were motivated to work for monetary rewards, they were explicitly informed that they would directly receive the accumulated monetary bonuses after the scan session. Further, they were asked to refrain from drinking any liquids for four hours prior to the start of the experiment time, to increase the chance that they would be thirsty. All participants indicated that they complied with instructions.

After completing the experiment session, participants filled out an exit questionnaire in which they indicated how strongly they liked the reward incentives, as well as how they liked the neutral solution. These ratings provide an index of how strongly individuals were motivated to perform when they were cued that the trial was a neutral trial (with the neutral solution delivered) or a reward trial (money or liquid incentive). These ratings were conducted on a Likert scale ranging from 1 (very strongly dislike) to 7 (very strongly like).

Visual stimuli were presented using PsyScope software [17] running on Apple PowerMac G4. Stimuli were projected to participants with an LCD projector onto a screen positioned at the head end of the bore. Participants viewed the screen through a mirror attached to the head coil. A fiber-optic, light-sensitive key press interfaced with the PsyScope Button Box was used to record participants' behavioral performance.

### Functional Imaging

Images were acquired on a head-only Siemens 3 Tesla Allegra System (Erlangen, Germany). A pillow and tape was used to minimize head movement in the head coil. Headphones dampened scanner noise and enabled communication with participants. Both structural and functional images were acquired at each scan. Anatomical images were acquired using a high-resolution MP-RAGE T1-weighted sequence. Functional images were acquired using an asymmetric spin-echo echo-planar sequence sensitive to blood-oxygen-level-dependent (BOLD) magnetic susceptibility (TR = 2.5 sec; TE = 25 msec; FA = 90 deg; slice thickness = 4 mm; in-plane resolution = 4×4 mm2; 32 slices). Functional images were acquired parallel to the anterior-posterior commissure line allowing complete brain coverage at a high signal-to-noise ratio [18]. Each scanning run consisted of two task blocks of 10 trials (each approximately 180 seconds duration) alternating with three fixation blocks (each 50 sec duration). During task blocks, the inter-trial interval was variable, from 2.5 seconds to 7.5 seconds in steps of 2.5 seconds (approximately 1/3 each of 2.5, 5, and 7.5 seconds), in order to create the necessary temporal jitter to allow deconvolution of event-related fMRI responses. The first four images in each scanning run were used to allow the scanner to ensure equilibrium of longitudinal magnetization, and were discarded.

### Liquid Setup

Juice was delivered using three digital infusion pumps (model SP200i, made by World Precision Instruments), which were adjustable to allow an exact amount of Liquid to be delivered. Each pump operated a 60 ml syringe (BD 60 ml syringe with Luer-Lok Tip, Fischer Scientific) filled with Liquid. Liquids were delivered through a length of Tygon tubing, which went from the machine, through the access port, to the scanner and into the participant's mouth. The pumps were linked to and triggered by PsyScope via a digital line-in port.

### Data Analysis

Behavioral data were analyzed for incentive effects by conducting ANOVAs and t-tests on error rates and reaction times (RT). Functional imaging data were pre-processed and statistically analyzed using in-house software. Pre-processing involved temporal alignment of volume slices (to correct for asynchronous slice acquisition), normalization within each scanning run to a fixed image intensity value, and then correction for motion using a rigid-body rotation and translation algorithm [19], [20]. Anatomical images were transformed into standardized atlas space [21], using a 12-dimensional affine transformation [22], [23]. The functional data were then resampled into 3 mm cubic voxels, registered to the subject's anatomical images, and spatially smoothed with a 9 mm FWHM (Full-Width, Half-Maximum) Gaussian kernel.

To assess both transient and sustained activity, a mixed blocked/event-related design was used, such that sustained or tonic activity could be dissociated from transient or trial-specific activation [24]. A general-linear model approach [25] was used to estimate parameter values for both event-related responses (item effects) and for sustained activity associated with the entire task block (state effects). State effects were independently coded into the GLM, using an assumption of a fixed-shape response of long duration (i.e., boxcar convolved with a gamma function [26] comprising the whole block). The logic of the GLM estimation approach is that event-related effects will decay back to Baseline during the ITI, while state-related effects should remain relatively constant, and of increased amplitude relative to control (fixation) blocks. This approach to GLM coding of sustained and transient responses has been validated via both simulation and empirically based methodological studies [24]. Additionally, in order to ensure that sustained effects occurring during task blocks were not confounded with the large transition effects in brain activity that are known to occur at the onset and offset of task blocks [27], separate GLM regressors coded for these transition periods. Event-related effects were analyzed by estimating values for the various time points within the hemodynamic response epoch. The duration of this epoch was taken to be 30 s (twelve scanning frames). This approach to GLM estimation (as opposed to a fit to predefined hemodynamic response function model) has been found to be critical in estimating event-related responses in mixed blocked/event-related designs [24]. Separate regressors coded for the different trial types (7 total: 3 each in the Liquid and Money, plus 1 in the Baseline condition). Additional regressors coded for trials in which errors were made.

An ROI-based approach was used to identify regions showing incentive effects. Two discrete networks were of interest (Note that hereafter the term ‘network’ does not refer to a functionally-connected network but is used instead to indicate a coherent set of regions assumed to be functionally related to reward processing and cognitive control). The first was a canonical network of brain regions engaged in cognitive control and WM, as defined by meta-analyses, which predominantly includes dorsal medial and lateral prefrontal and parietal regions [28], [29]. The second was the core network of brain regions associated with reward and affect, which primarily includes subcortical regions such as the ventral and dorsal striatum and amygdala, but also cortical structures such as the posterior insula and ventral/orbital PFC (see Knutson, 2003 amongst others). To define the regions included in the cognitive control network (CCN), we created a mask of spherical ROIs (10 mm radius) using anatomical coordinates of regions described in two published meta-analyses as seed points [28], [29]. We have used this identical mask successfully in prior published studies [30], [31]. The regions included in the reward network (REW: amygdala, nucleus accumbens, putamen, caudate nucleus, substantia nigra, ventromedial PFC, insula, orbitofrontal cortex), were hand-drawn according to anatomical landmarks as well as coordinates provided in previously published studies of reward effects [13], [32]–[39]. The exact masks and coordinates for both networks are available from the authors by request.

These ROI masks were used to constrain analysis to only those voxels that are theoretically most strongly associated with working memory and reward processing. We then identified voxel clusters from within these masks that showed particular incentive effects of interest. We were interested in examining both common and category-selective patterns of incentive effects that were present in both sustained and transient activation components. To identify each of these patterns we constructed multiple contrasts, and a voxel cluster was only identified if it simultaneously satisfied all of the contrasts (p<.05, uncorrected; minimum cluster size  = 8 voxels). For the sustained activity patterns, the contrasts involved the sustained GLM estimates in each of the three different conditions. For transient patterns, the contrasts involved the GLM estimates for the different trial types, averaged across time-points 2–6, which captures the period during which the incentive cue is presented and task performed (after accounting for the approximate 3–6 second hemodynamic lag), but likely minimizes effects related to reward feedback. The following contrasts were used for *sustained* activity effects: 1) Common  =  Money > Fixation, Money > Baseline, Liquid > Fixation, and Liquid > Baseline; 2) Money-Selective  =  Money > Fixation, Money > Baseline, and Money > Liquid; 3) Liquid Selective  =  Liquid > Fixation, Liquid> Baseline, and Liquid > Money. The following contrasts were used for *transient* activity effects: 1) Common  =  Money HI > Fixation, Money HI > Baseline NO, Money HI > Money NO, Liquid HI > Fixation, Liquid HI > Baseline NO, Liquid HI > Liquid NO; 2) Money Selective  =  Money HI > Fixation, Money HI > Baseline NO, Money HI > Money NO, Money HI > Liquid HI; 3) Liquid Selective  =  Liquid HI > Fixation, Liquid HI > Baseline NO, Liquid HI > Liquid NO, and Liquid HI > Money HI.

Finally, a supplementary whole-brain exploratory analysis was also conducted to exclude the possibility of overlooking significant activation in regions other than the specified ROIs. The same contrasts were used, but each t-test was FDR corrected for multiple comparisons (p<0.05) first. In a second step, these corrected contrasts were included in the identical conjunction described for the ROI analysis. This more stringent identification procedure was employed for the whole-brain analysis since it was exploratory and therefore more prone to false positives.

## Results

### Behavioral Results

#### Salience of reward incentives

Analyses of the subjective liking ratings of the money and liquid incentives, as well as the neutral solution were collected to verify the validity of the incentives as rewarding or neutral outcomes. Money incentives were liked significantly compared to a neutral rating of “4” (Low: M = 6.45, SD = .78, *t*(30) = 10.29, p<.001; High: M = 6.71, SD = .64 *t*(30) = 12.72, p<.001), and juice incentives were significantly more liked upon receipt than the neutral liquid (Low: M = 5.73, SD = 1.19, *t*(60) = 8.236, p<.001; High: M = 5.90, SD = 1.22 *t*(60) = 8.685, p<.001). Importantly, the rating of the neutral liquid was indeed neutral, as the mean rating score did not differ significantly from the expected neutral rating of “4” as defined by our scale (M = 4.24, SD = 1.70, *t*(60) = .638, n.s.). Thus, the incentives that we offered were truly rewarding, and our neutral liquid truly neutral.

#### Response times (RT)

Analysis of RTs only considered correct trials. We first examined blocked incentive effects. A one-way repeated-measures ANOVA revealed a significant main effect of incentive condition (Baseline, Money, Liquid; F(1,30) = 147.12, p<0.001). This was due to a significant difference between RTs in the Baseline condition (946 ms) and each of the two incentive conditions (Baseline vs. Money: Mean: 714 ms, t(30) = 10.65, p<0.001; Baseline vs. Liquid: Mean: 705 ms, t(30) = 11.75, p<0.001). The two incentive conditions did not differ in RT (t(30) = 0.57, p = 0.573).

**Figure 2:**
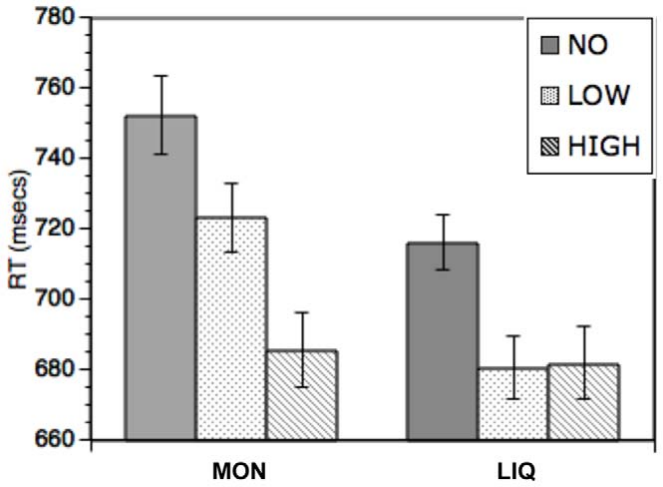
Trial-specific incentive effects. Response times for no-incentive trials (NO), low incentive trials (LOW) and high incentive trials (HIGH) for the Money (MON) and Liquid (LIQ) blocks. Figure demonstrates main effect of incentive magnitude, reflected in faster response times on trials with higher incentive value.

To examine trial specific effects, only trials from the incentive blocks were included. A 2-way (2×3) ANOVA including the factors category (Money vs. Liquid) and magnitude (high vs. low vs. no-incentive trials) revealed a significant main effect of incentive magnitude (F(2,29) = 20.56, p<0.001, Figure 2) demonstrating faster RT for incentive trials compared to no-incentive trials. Additionally, faster RTs in high incentive compared to low incentive trials were observed (t(30) = 4.22, p<0.001). Nevertheless, there was a marginally significant magnitude X category interaction (F(2,29) = 2.82, p = 0.067). This interaction was due to the fact that the magnitude effect for low vs. high incentive trials was observed in the Money condition, but not in the Liquid condition (Money: t(30) = 4.59, p<0.001; Liquid: t(30) = 1.25, p = 0.221).

#### Error rates

Overall error rates for all trials were low (Baseline  = 3.1%, Money  = 3.7%, Liquid  = 2.6%). There was no significant effect of block type on error rates (F(2,25) = 1.02, p = 0.368). Likewise looking only at error rates among incentive trials, the effects of magnitude, category, and their interaction were all insignificant (p's >.06). Together, these results indicate the absence of any speed-accuracy tradeoff, and provide strong support for the idea that WM performance was improved under incentive conditions.

### Imaging Data: ROI Approach

#### Sustained effects

**Figure 3:**
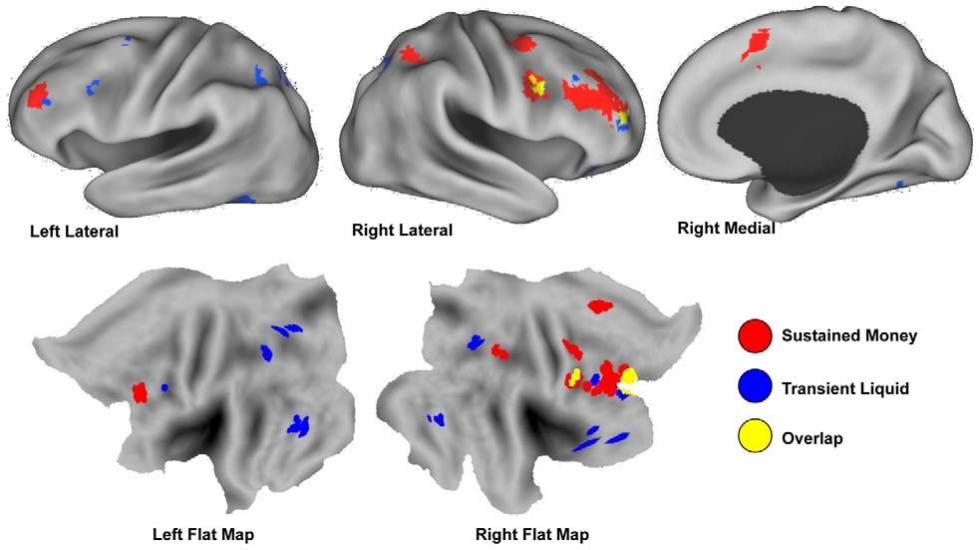
Incentive category specific activation and overlap. Regions showing selective transient incentive effects in Liquid (blue) and selective sustained incentive effects in Money (red). Regions showing a shift from transient activation during the Liquid condition to sustained activation during the Money condition (i.e., overlap regions) are shown in yellow.

Six regions within the cognitive control network (CCN) showed sustained activity increases selectively in the Money incentive condition (Figure 3, red regions; see also Table 1). These were primarily right-lateralized and included: anterior prefrontal cortex (aPFC), dorsolateral prefrontal cortex (dlPFC), anterior cingulate cortex (ACC) and inferior parietal cortex. These regions replicate well the regions identified to show monetary incentive-related sustained activity in our prior work [8], even though that study involved a different task, no trial-by-trial incentive manipulation, and no comparison to Liquid incentives. Additionally, within the reward processing network, Money-selective sustained activity was observed in the head of the right caudate.

**Table 1:** Sustained and transient effects for the cognitive control and reward processing networks.

	Network	Incentive pattern	Volume	BA	x	y	z
**Sustained Effects**							
Left Anterior PFC	CCN	MON selective	945	10	−34	40	18
Right anterior/DLPFC	CCN	MON selective	5670	46/10	35	36	22
Right DLPFC/IFJ	CCN	MON selective	1971	44/9	43	6	30
Right Premotor Cortex	CCN	MON selective	1728	6	33	0	49
Dorsal Anterior Cingulate	CCN	MON selective	1269	6	4	8	50
Right Parietal Cortex	CCN	MON selective	756	40	32	−49	44
Left Anterior PFC	CCN	Common	297	46	−44	41	14
Superior Temporal Cortex	CCN	Common	243	22	−63	−3	8
Right Parietal Cortex	CCN	Common	945	7	36	−62	46
Dorsal Striatum	REW	MON selective	729	---	10	−2	19
Left Insula	REW	MON selective	405	13	−32	9	11
Left Amygdala	REW	LIQ selective	486	---	−18	−8	-18
Right Amygdala	REW	LIQ selective	297	---	18	−8	−20
Right Ventral Striatum	REW	LIQ selective	351	---	7	0	−4
*Dorsal Striatum*	REW	*LIQ selective*	*135*	*---*	*−9*	*12*	*3*
*Right Lateral OFC*	REW	*Common*	*108*	*11*	*35*	*21*	*−9*
*Dorsal Striatum*	REW	*Common*	*108*	*---*	*9*	*8*	*6*
**Transient Effects**							
Right Anterior PFC	CCN	LIQ selective	1485	10	34	49	14
Right DLPFC	CCN	LIQ selective	891	9	41	35	34
Left VLPFC	CCN	LIQ selective	243	45	−33	33	16
OFC	CCN	LIQ selective	1269	11	34	34	−10
Right DLPFC/IFJ	CCN	LIQ selective	810	9	43	7	31
Left DLPFC/IFJ	CCN	LIQ selective	270	9	−46	11	30
Left Premotor Cortex	CCN	LIQ selective	270	6	−34	3	58
Left Superior Parietal Cortex	CCN	LIQ selective	486	7	−19	−71	43
Left Superior Parietal Cortex	CCN	LIQ selective	432	7	−31	−58	43
Right Parietal Cortex	CCN	LIQ selective	378	7	23	−64	43
Cerebellum	CCN	LIQ selective	351	---	34	−55	−18
Cerebellum	CCN	LIQ selective	540	---	4	−71	−20
Cerebellum	CCN	LIQ selective	270	---	0	−61	−26
Cerebellum	CCN	LIQ selective	2295	---	−39	−58	−19
Right Lateral OFC	REW	LIQ selective	2835	11	30	36	−11
Left Lateral OFC	REW	LIQ selective	2646	10	−16	35	−12
Medial OFC	REW	LIQ selective	513	11	7	47	−20
*Medial OFC*	REW	*LIQ selective*	*135*	*11*	*11*	*17*	*−21*

1 Cluster sizes in mm^3^ and talairach coordinates are shown.

2 Regions of a cluster size smaller than 8 voxels are printed in italic.

Sustained effects were found specific to the Money blocks, specific to the Liquid blocks, and common to both incentive conditions. Transient effects were found specifically for the Liquid blocks. No transient effects were found selective to Money or common for both Money and Liquid.

In contrast, for the Liquid condition, there were no regions showing selective sustained activity in the CCN, but in the reward processing network (REW), selective sustained activity was observed in four regions: bilateral amygdala, and the left dorsal and right ventral striatum, with the latter matching well the anatomical location of the nucleus accumbens.

Finally, sustained activation common to both incentive conditions was observed in left inferior frontal cortex, left anterior PFC and right parietal cortex within the CCN. With a lowered cluster size, additional common incentive effects in the REW network were observed in the left dorsal striatum and right lateral OFC.

#### Transient effects

We next examined event-related increases related to the trial-by-trial incentive manipulation. In contrast to the patterns observed with regard to sustained activity, for the Money condition, no incentive-related transient activation increases were observed in the CCN, while a large number of regions in this network showed selective effects in the Liquid condition. These included bilateral ventrolateral prefrontal cortex (vlPFC) and dlPFC, right anterior PFC bilateral inferior parietal cortex and medial cerebellum (Figure 3, blue regions). Additionally, in the REW network selective transient increases were observed in lateral and medial orbitofrontal cortex (OFC) for the Liquid condition (Table 1 provides a full list of regions). There were no additional regions in the CCN or REW network showing transient effects that were common to both incentive conditions.

The lack of transient activation increases in the Money condition was surprising given that such effects have been previously observed in both motivation and reward tasks using monetary incentives. For example, in studies with the monetary incentive delay task, reward incentives were associated with transient increases in activation within subcortical regions such as the ventral striatum [13], [37]. However, these studies have typically employed less stringent activation contrasts than the ones employed here, which tested for selective or common effects across multiple incentive conditions, and also required increased activation relative to baseline (i.e., no-incentive) blocks. Thus, to more properly compare our results to these prior studies we conducted an additional analysis focusing only on the Money condition, and contrasting only the high and no-incentive trials. This less stringent contrast revealed increased incentive-related transient activation in the CCN network within the right anterior PFC, anterior insula, cerebellum and thalamus, and in the REW network within the bilateral dorsal striatum (caudate nucleus), right ventral striatum and left amygdala (all p's <0.05, observed in a priori ROIs; a full list of regions and coordinates will be sent upon request). Thus, with less stringent contrasts the results do not depart markedly from the transient effects found in prior studies using monetary incentives.

#### REW-CCN double dissociation

**Figure 4:**
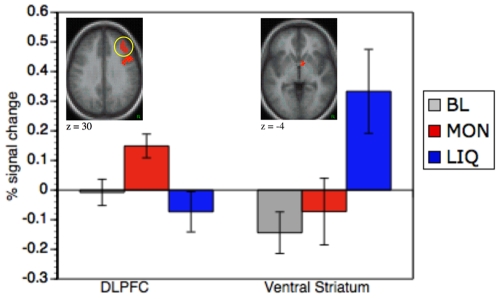
Anatomical double dissociation in incentive category specific sustained activation. Sustained activation selective to the Liquid condition was observed in the reward network as representatively shown for the ventral striatum (7, 0, −4), whereas the cognitive control network showed money-selective state effects (here shown for the DLPFC (35, 36, 22).

The results described above suggest an anatomical dissociation in sustained activity between components of the brain's cognitive control and reward networks. Specifically, CCN regions (e.g., right lateral PFC and parietal cortex) showed selective sustained increases during the Money condition, whereas REW regions (e.g., ventral striatum and amygdala) showed selective sustained increases during the Liquid condition. To statistically confirm this pattern, we ran 24 2-way ANOVAs including the factor region (cognitive control region vs. reward region) and category (Money vs. Liquid) to test for interactions between region and category on magnitude percent signal changes in the isolated regions. Every cognitive-control-related region was tested together with each reward-related region in a separate ANOVA. Each ANOVA revealed a significant cross-over interaction effect between category and region (all p's<0.05). Figure 4 shows this pattern for a representative pair of regions – right dorsolateral PFC and left ventral striatum.

#### Overlap effects

**Figure 5:**
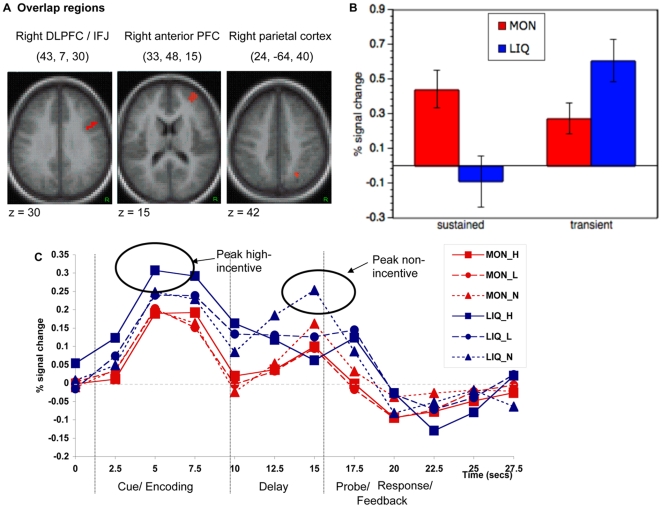
Flexibility in activation dynamics of cognitive control regions related to incentive magnitude and category. A) Overlapping regions showing selective state effects in Money and selective item effects in Liquid. B) Overlapping regions showing a shift from sustained to transient activation across the Money and Liquid incentive conditions. Percent signal change average for all three overlapping regions, sustained effects showing averaged signal changes across the incentive block, and transient effects are averaged across frames 2 to 6 in high incentive trials. C) Time-courses for incentive trials and no-incentive trials within the Money and Liquid condition showing a shift in the peak of activation dynamics during the Liquid condition. On no-incentive trials, activation peaks late in the trial (around probe response), and on incentive trials, activation peaks earlier (during encoding). All are averaged for the three overlapping cognitive control regions. (MON_H: money high-incentive trials, MON_L: money low-incentive trials, MON_N: no-incentive trials during the Money block; LIQ_H: liquid high-incentive trials, LIQ_L: liquid low-incentive trials, LIQ_N: no-incentive trials during the Liquid block).

Within the CCN, a striking finding was that in the Money conditions sustained activation increases tended to predominate, whereas in the Liquid condition a transient pattern of activation was observed. We tested whether any regions showed both patterns of activity by conducting a direct overlap analysis of money-selective state effects and liquid-selective transient effects. Three regions of overlap were observed in anterior PFC, lateral/posterior PFC (including the inferior frontal junction; IFJ) and posterior parietal cortex, all in the right hemisphere (Figure 3 yellow regions; see also Table 2 and Figure 5A). We confirmed that these regions showed a shift from sustained to transient activation across the Money and Liquid incentive conditions, through an ANOVA with region, category (Money vs. Liquid) and dynamics (sustained, event-related) as factors. A significant category X dynamics interaction was observed (F(1,30) = 9.84, p<0.01, Figure 5B), with no further interaction across the different regions (region X category X dynamics: F(2,29) = 2.840, p>.05). The interaction was of the cross-over form, with sustained activity higher in the Money condition, but transient activity higher in Liquid.

**Table 2:** Regions showing selective state incentive effects in the money and selective item incentive effects in the liquid condition.

Region	Brodmann area	size (mm^3^)	x	y	z
Right Anterior PFC	10	702	+33	+48	+15
Right DLPFC/IFJ	44/9	783	+43	+7	+30
*Right parietal cortex*	*7*	*135*	*+24*	*−64*	*+40*

1 Regions of a cluster size smaller than 8 voxels are printed in italic.

#### Timecourse analyses

The transient activation effects observed within CCN regions engaged during the Liquid condition suggest a distinct form of cognitive control from that engaged during the Money condition. We further examined this issue through focused analyses of the timecourse of activity in each condition for the three overlapping regions. For these regions showing money-selective state effects and liquid-selective transient effects, a prominent pattern during the Liquid condition was not just increased activity on incentive vs. no-incentive trials, but also a shift in the peak of activation dynamics. On no-incentive trials activation peaked late in the trial, presumably around the time of the response and feedback. In contrast, on incentive trials, activity peaked much earlier, presumably during the encoding or delay period (see Figure 5C). This shift in activation dynamics reflects a cross-over pattern with an incentive > no-incentive pattern in the early part of the trial, but a no-incentive > incentive pattern in the late part of the trial. This cross-over dynamic is consistent with a shift to proactive control (engaged during encoding and WM maintenance periods) from reactive control (engaged primarily during response periods) that we have observed in prior work. A trial-type (high, no-incentive) X period (early: average of time-points 3,4; late: average of time-points 6,7) X incentive category (money, liquid) interaction (F(1,30) = 8.15, p = 0.01) statistically confirmed this cross-over pattern being selective for the Liquid condition, but not present during the Money condition. This was also supported by post-hoc comparisons (Liquid: early-HI vs. early-NO, t(30) = 2.33, p<0.05; late-HI vs. late-NO, t(30) = -6.42, p<0.05; Money: early-HI vs. early-NO, t(30) = 0.7, n.s.; late-HI vs. late-NO, t(30) = −1.5, n.s.).

Moreover, inspection of the other CCN regions showing Liquid-selective effects revealed the same cross-over pattern in activation dynamics was observed during the liquid condition, with activation shifting from a late peak on no-incentive trials to an early peak on incentive trials. A significant interaction and no main effects of trial-type or magnitude (consistent with a cross-over pattern) were observed for five regions including bilateral dlPFC, bilateral anterior PFC, and left parietal cortex.

#### Sustained-transient relationship

**Figure 6:**
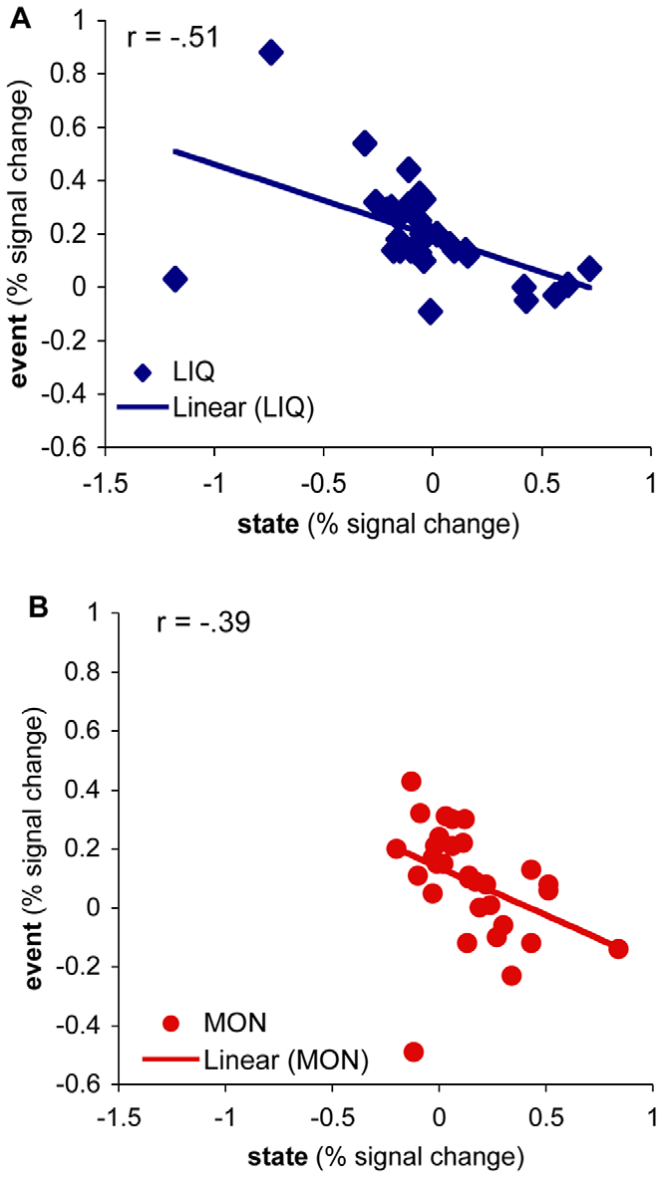
Correlations between sustained and event related BOLD responses. Percent signal changes are averaged for the overlapping regions. Sustained and event related activations showed significant correlations in both the Liquid condition (A) and the Money condition (B).

The relationship between sustained and transient activation dynamics is an important pattern observed in both incentive conditions. In the Money condition, a dominant pattern was an increase in sustained activation and a tendency for transient activation to decrease (relative to Baseline). Conversely, in Liquid the opposite pattern was present (decrease in sustained activation, plus increase in transient activation relative to Baseline). We tested whether these two patterns were related by examining the association between sustained and transient activation patterns across subjects. In the three overlapping regions, a negative correlation between transient activity (averaged across time points 2 to 6) and sustained activation was observed not only in the Liquid condition but also in Money (Money: r = −.39, p<0.05, Liquid: r = −.51, p<0.01, see Figure 6). This pattern replicates that observed in a prior analysis of the Money condition for this dataset [40], and suggests that the changes in transient and sustained activity may be linked. That is, the increased sustained activation found in Money may produce a corresponding decrease in transient activation during this condition. Conversely, the transient activation found in Liquid may be due to the absence of sustained activation changes in this condition.

#### Performance – activation relationship

**Figure 7:**
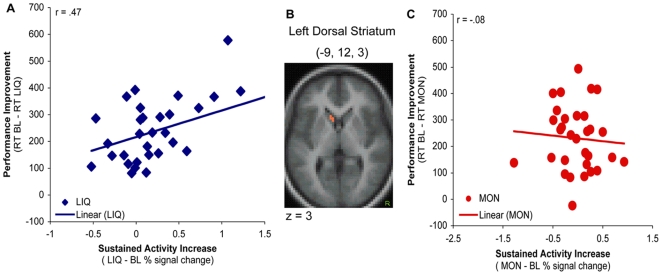
Activation-performance relationship in the dorsal striatum. Correlation between sustained activation increase and performance improvement relative to baseline observed in the Liquid condition (A) within a region in the left dorsal striatum (−9, 12, 3; panel B). During the Money condition, no such correlation was present (C). Activation effect is percent change signal increase in incentive condition relative to Baseline. Performance effect is response time improvement (in msec) during incentive condition relative to Baseline.

The observed anatomical dissociation in sustained activity between components of the brain's cognitive control and reward networks suggest different mechanisms by which the two types of incentives modulate behavior. In a prior analysis involving this same dataset [40] we found that performance during monetary incentive blocks was mediated by both sustained and transient activation in cognitive control regions. Interestingly, the relationship was observed in a right dorsolateral PFC region, which was very close to right dlPFC showing money selective tonic effects. Given these results, we tested whether liquid incentive specific tonic activation in reward-related regions also predicted performance. Interestingly, the tonic activation increase in the left dorsal striatum (−9, 12, 3) region that was observed to be specific to the Liquid condition was also correlated to larger performance improvement in the Liquid block (i.e., faster RTs in Liquid vs. Baseline was associated with a larger increases in sustained activity in Liquid vs. Baseline; r = −.47, p<0.01; see Figure 7). This correlation was observed only in the Liquid condition but not in the Money condition (BOLD_MON – BOLD_BL; RT_BL – RT_MON; r = .08, ns) and did not occur in the right dorsal striatum region that showed sustained increases common to both condition (Money: r = .08, n.s.; Liquid: r = −.01, n.s.).

### Imaging Data: Exploratory Whole-Brain Analysis

A final exploratory analysis investigated whether any regions outside the CCN and REW networks showed either sustained or transient effects. No additional regions were identified that achieved statistical significance using whole-brain multiple comparisons correction.

## Discussion

The goal of the current imaging study was to investigate the mechanisms by which motivational incentives (primary and secondary rewards) influence cognitive control. The results support two key conclusions. First, they provide new evidence for the flexibility of cognitive control regions, supporting the idea of a functional dissociation of cognitive control based on temporal dynamics. Particularly in the primary incentive (Liquid) condition, we observed that changes in trial reward value were associated with a shift in event-related activation dynamics, from a late-peaking (during the response/feedback period) to an early-peaking (during the encoding period) profile. Interestingly, within those same brain regions, activation showed a shift from transient responses during the Liquid condition to sustained responses during the Money condition. Second, while behavioral performance was significantly and equally improved by both primary and secondary incentives, each incentive type selectively impacted anatomically dissociable regions. Secondary (Money) incentives were associated with selective increases in sustained activity within a network of cognitive control regions including bilateral anterior PFC and right lateralized dlPFC, parietal cortex and ACC. Conversely, primary (Liquid) incentives were associated with selective sustained activity increases in subcortical reward regions, including the amygdala and striatum. Each of these findings yields important new information regarding the neural mechanisms underlying motivation-cognition interactions, which are discussed in detail in the following sections.

### Temporal Dynamics of Motivated Cognitive Control

Overall, these results present new evidence for the flexible nature of activity dynamics within cognitive control regions, as well as support the idea of a dissociation of cognitive control based on temporal dynamics [41]. In prior work, we have put forward the Dual Mechanisms of Control (DMC) account, which suggests a dissociation between two modes of cognitive control: a proactive mode, in which control is engaged in an anticipatory/sustained fashion during encoding and maintenance periods, and a reactive mode, in which control is engaged in a just-in-time manner, during probe/response periods [42]. An important component of the DMC account is that these control modes can be flexibly engaged within the same anatomical regions, via a shift in the activation dynamics of engagement. Thus, the DMC account has a different emphasis from (but is not in conflict with) anatomically based models that assume modulations in control processes require differential selection of anatomically segregated regions [43].

In the current study, we observed a cross-over pattern in activation dynamics in prefrontal regions including lateral and anterior PFC and parietal cortex during the Liquid incentive condition, as evidenced by an increase of activation during the encoding phase of incentive trials (representing a proactive cognitive control process) relative to stronger responses at the retrieval phase of no-incentive trials (representing a reactive cognitive control process). This shift from reactive control during no-incentive trials to proactive control during incentive trials was observed in conjunction with behavioral improvement in incentive trials. This provides evidence for a functional dissociation of cognitive control in common regions due to temporal dynamics. Specifically in this task, the observed shift from reactive control during no-incentive trials to proactive control during incentive trials likely reflects a change in mnemonic strategies to enhance performance. During incentive trials, the increased value of task goals may be encoded in PFC regions to facilitate a more effective updating of working memory during the encoding period [44]. This would maximize the chance of receiving a reward, whereas such a strategy may not have been implemented during the no-incentive trials.

Interestingly, our results also extend the idea of flexible activation dynamics in lateral PFC and parietal cortex from trial-by-trial variations to a block-level distinction between sustained and transient mechanisms. In those same regions (dlPFC, vlPFC and parietal cortex), we additionally observed a shift from a transient activation pattern during the liquid incentive blocks to a sustained pattern of activation during the monetary incentive blocks. This cross-over pattern in activation dynamics suggests that the different categories of incentives instantiate different operational modes in the same cognitive control regions. We suggest that this pattern is also consistent with a different type of proactive control shift. In particular, the sustained activation of these regions across trials may provide a means of maintaining the increased goal-value of trials within the Money block, such that this value does not have to be re-encoded with each new trial. One speculative hypothesis, that we discuss further below is that a sustained encoding and maintenance of task goal value may be more important in the Money block, because in this block the incentive cues themselves may not be as effective in conveying the relevant trial reward value.

Regardless of the particular interpretation given for the present results, it is clear that that motivational incentives can lead to performance enhancements; our results suggest that this behavioral effect is due to a shift in activation dynamics towards earlier and more sustained patterns. Overall, the variety of dynamical patterns observed in lateral PFC (i.e., shifts from transient to sustained, and reactive to proactive) during the same task can be considered strong evidence for the involvement of a fluctuating combination of context-dependent neuronal control mechanisms employed to perform demanding cognitive tasks [41].

### Incentive Category Effects

To our knowledge, this study represents the first time that the neural correlates of different types of reward incentives have been examined using a within-subject design. The results suggest that at least a component of the neural mechanism of reward incentive processing might be category dependent. During the Money condition, cognitive control regions showed mainly right lateralized sustained activation patterns, whereas the Liquid condition was marked by selective transient effects that extended to left lateralized regions including anterior PFC, dlPFC, and parietal cortex. Further, a double dissociation of sustained neural activation patterns was distinguishable between both primary and secondary incentive conditions. Tonic activation of cognitive control regions during the monetary incentive blocks was observed in bilateral anterior PFC and right lateralized dlPFC, parietal cortex and ACC. In contrast, tonic neural activity in subcortical structures associated with reward processing – the bilateral amygdala and ventral striatum – was observed in liquid incentive blocks. This double dissociation suggests that although primary and secondary incentives improve behavioral performance to an equivalent degree, they exert their benefits through different neural mechanisms and reinforcement processes.

One might speculate based on these results that the Liquid condition served as a special case of reward processing. However, we would argue that instead it may be the reverse – that specialized neuronal mechanisms are recruited for processing monetary incentives. Monetary incentives serve as a more abstract reward, since reward delivery was delayed until after the completion of the task, whereas in the liquid condition the reward was delivered immediately and consumed as a direct behavioral consequence of performance. The sustained activation in cognitive control regions during the money condition may have served to maintain a representation of the increased value or salience of task performance, in the absence of the direct hedonic experience of the reward incentives during task trials. Thus, the Money condition can be conceptualized as including a delay of gratification component, since cognitive effort had to be consistently and continually applied in order to receive a delayed, accumulated payoff as opposed to directly experiencing a tangible reward on each trial [45], [46].

In contrast, the liquid incentives acted as an immediate reinforcer providing a concrete external reward, which elicited activation in the bilateral striatum and amygdala. These regions are key components of a core affective-motivational system involved in learning new behavioral responses to cues paired with reinforcers [47], [48]. In this system, projections arising from the midbrain dopamine (DA) system are thought to provide a learning signal that enables associative transfer of the reinforcer value to cues that reliably precede it [49]. Over time, these cues gain incentive salience and elicit DA responses similar to those induced by an actual reward. Additionally, according to Berridge [50], [51] incentive cues themselves become “motivational magnets”, triggering a ‘wanting’ response and energizing approach behavior. The sustained activation observed in the striatum and amygdala in the Liquid condition may have thus reflected a tonic representation of incentive salience and increased motivational drive that emerged following repeated exposure to primary reinforcers and the associated instrumental learning process.

The differential engagement of lateral PFC in the Liquid and Money condition might also be related to the role of reward-related DA activity. In particular, in prior work we have argued that phasic DA responses occurring to task cues may serve a gating function within lateral PFC, enabling the effective updating and maintenance of PFC-mediated task goal representations [52], [53]. Thus, the increased early transient activation observed in lateral PFC during the Liquid condition is consistent with a pattern that would be predicted if the incentive cues on reward trials were associated with an increased phasic DA response, arising via the reinforcement learning process described above. In contrast, during the Money condition, incentive cues may not have triggered a phasic DA response, because the process of reinforcement learning (i.e., transfer of incentive salience from the reward feedback period to the time of the incentive cue) was less effective with abstract (i.e., visual, symbolic) rather than primary rewards. Instead, in the Money condition, it may have been necessary to maintain a cognitive representation in working memory of the accumulated reward value and integrate this value with task goal-related information in order to prevent performances lapses across the task block. The sustained activation of lateral PFC may have reflected the tonic maintenance of reward value in working memory across trials. This hypothesis, though speculative, is consistent with the prior literature. A common finding is that lateral PFC regions do show sustained activation patterns that are associated with goal maintenance functions and the top-down biasing of goal-driven behaviors [54].

This interpretation of the differential effects of incentive category on neural activity and behavior is admittedly speculative, and will need to be tested with further direct investigations. Our interpretation suggests that the liquid and monetary reward cues should carry differential incentive salience to participants. This could be tested through psychophysiological measures that are thought to more directly index conditioning effects, such as pupil dilation, heart rate and skin conductance. A strong prediction is that conditioning-type effects should be observed more strongly to cues in the Liquid compared to Money condition, at least early on during task performance. However, an alternative interpretation of the effects is that they reflect the direct and immediate receipt of rewards in the Liquid condition, rather than something about the category of reinforcer per se. It could be possible to test this hypothesis, by delaying reward receipt in the Liquid condition (e.g., accumulating rewards across trials and delivering at the end of the block).

An additional direction of future investigation would be to provide support for differential causal pathways linking reward and cognition in the Money and Liquid conditions, through functional and effective connectivity analyses. In particular, one hypothesis is that the shifting temporal dynamics of cognitive control region activation across the Money (sustained) and Liquid (transient) conditions might be mediated by differential sustained activity in subcortical reward regions. Thus, for example, the transient increases in activity observed within lateral PFC and parietal cortex during the Liquid condition may be driven via sustained activation effects in striatum and amygdala during this condition. Although it has been difficult in the past to test complex mediational hypothesis, new tools that have recently become available might make such analyses possible in future work [55].

### Conclusions

The current results indicate that primary and secondary reinforcers are equally effective as motivational incentives in enhancing performance even during demanding cognitive tasks such as working memory. However, the findings also suggest that these different incentive categories may exert their effects by modulating cognitive control via dissociable neural mechanisms, both in terms of a shift between transient and sustained activation dynamics in lateral PFC and parietal cortex, and between a shift in sustained activity between the frontoparietal cognitive control network and subcortical reward network. We hypothesize that these differential neuronal mechanisms may reflect the distinction between motivational processes engaged through a core instrumental conditioning process triggered by direct receipt of primary rewards, such as juice, and a more cognitive process that helps to interpret delayed and symbolic rewards, such as money. However, this hypothesis will require more direct support obtained through further investigation. Nevertheless, the current results highlight the importance of examining primary rewards as well as secondary ones in studies of motivation and cognition, as well as in focusing on the temporal dynamics of brain activation when investigating the neural mechanisms of cognitive control.
